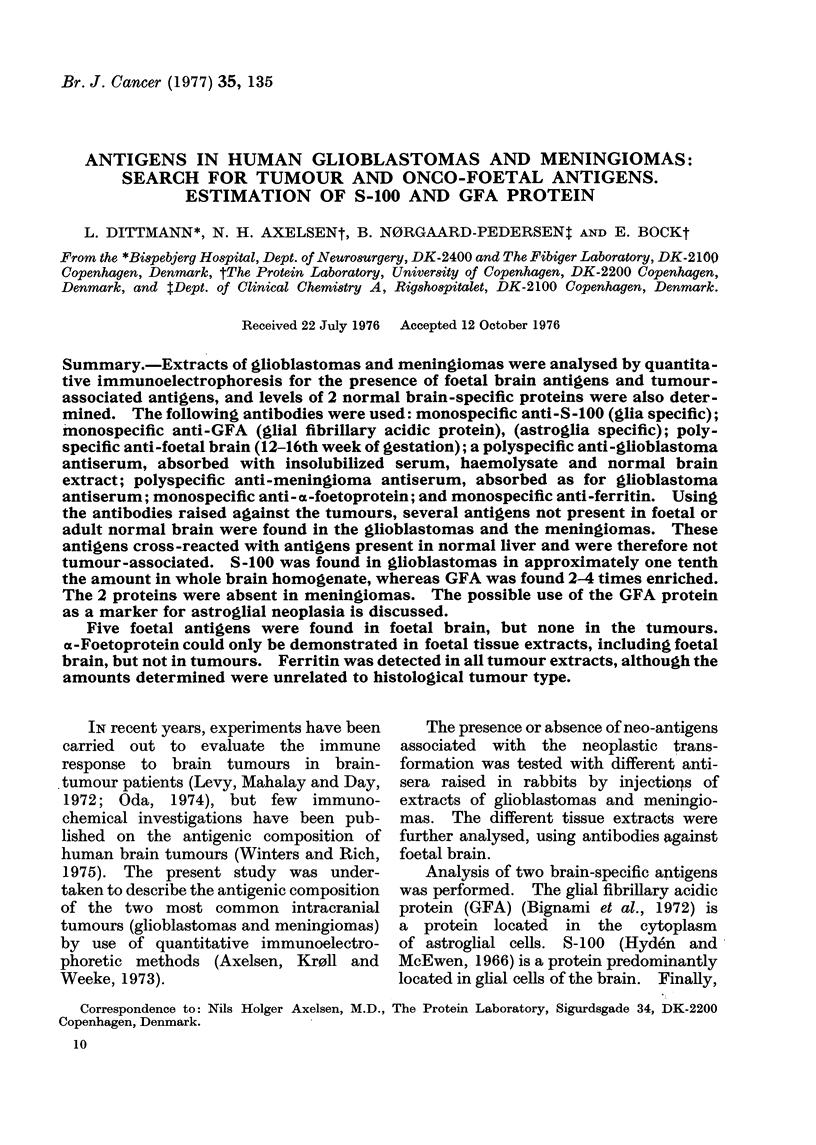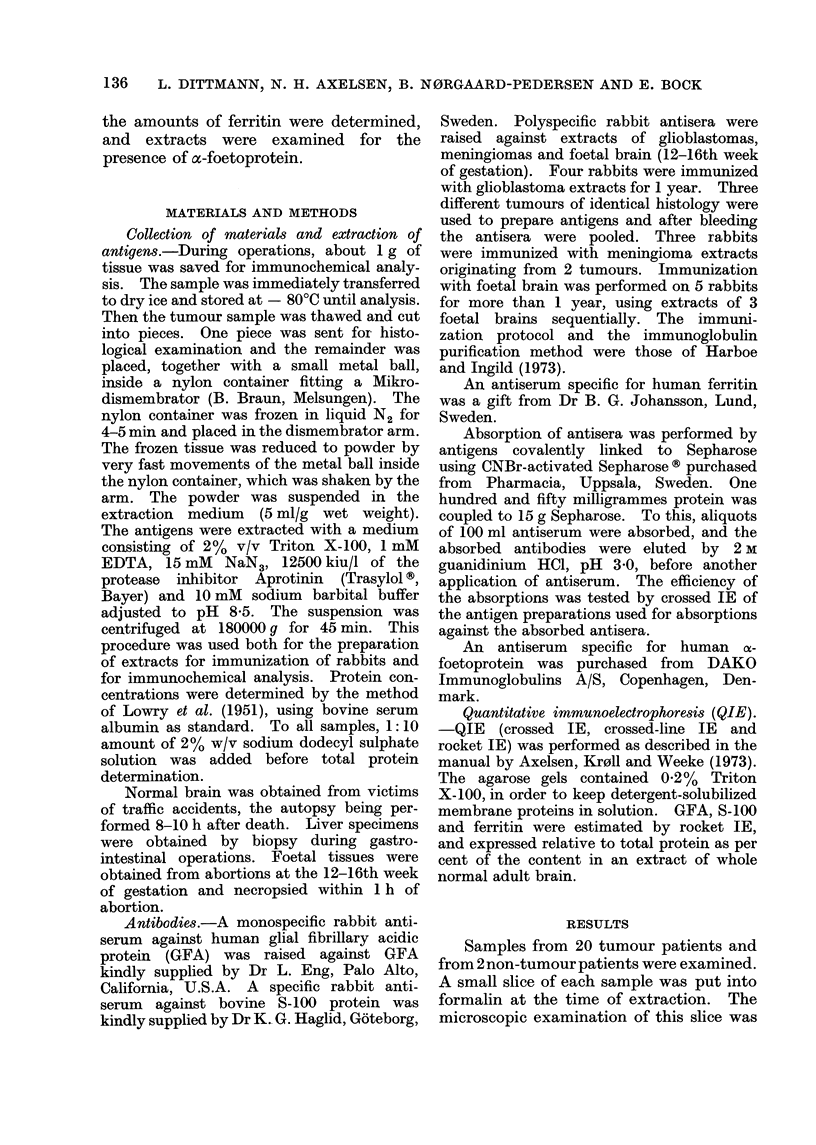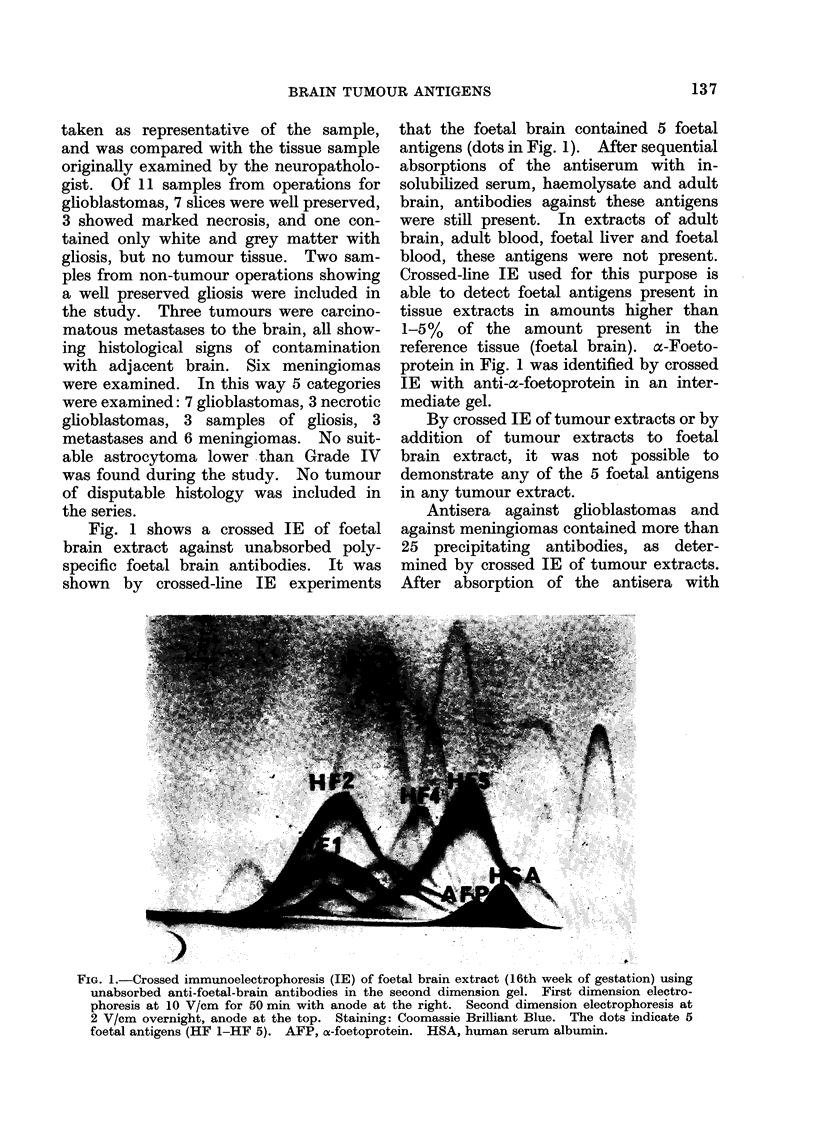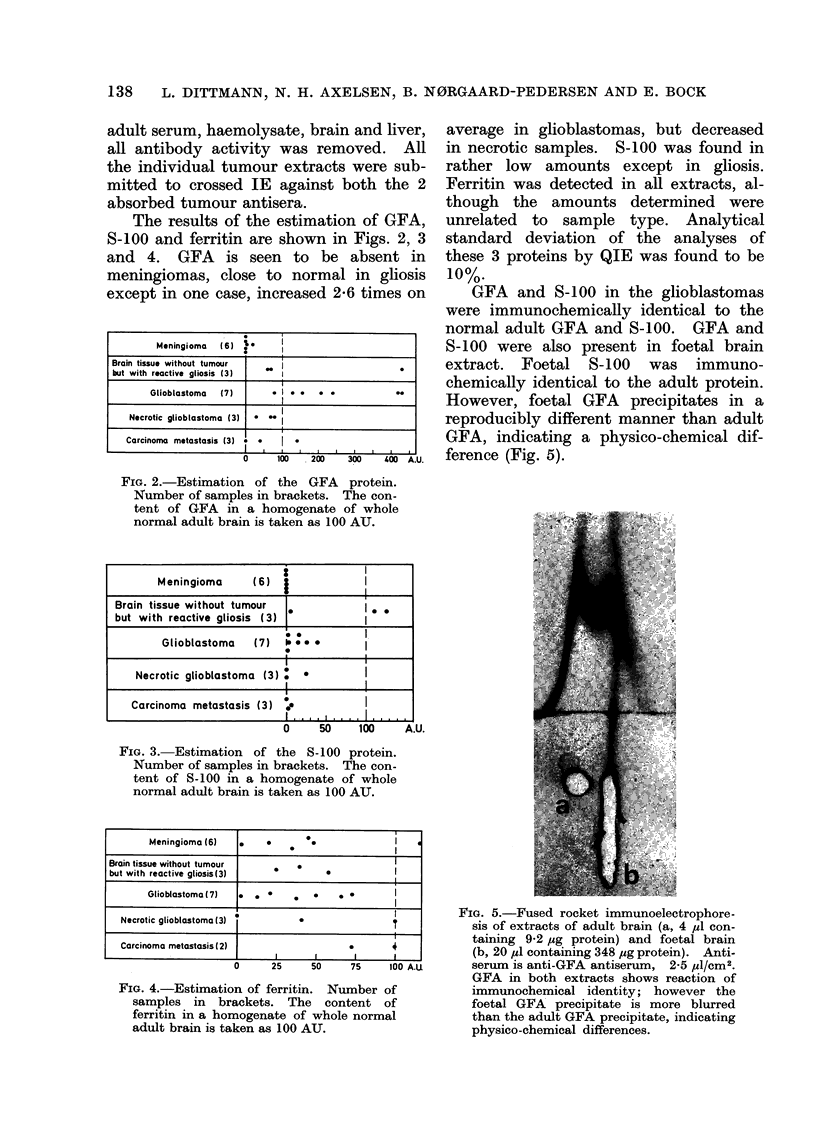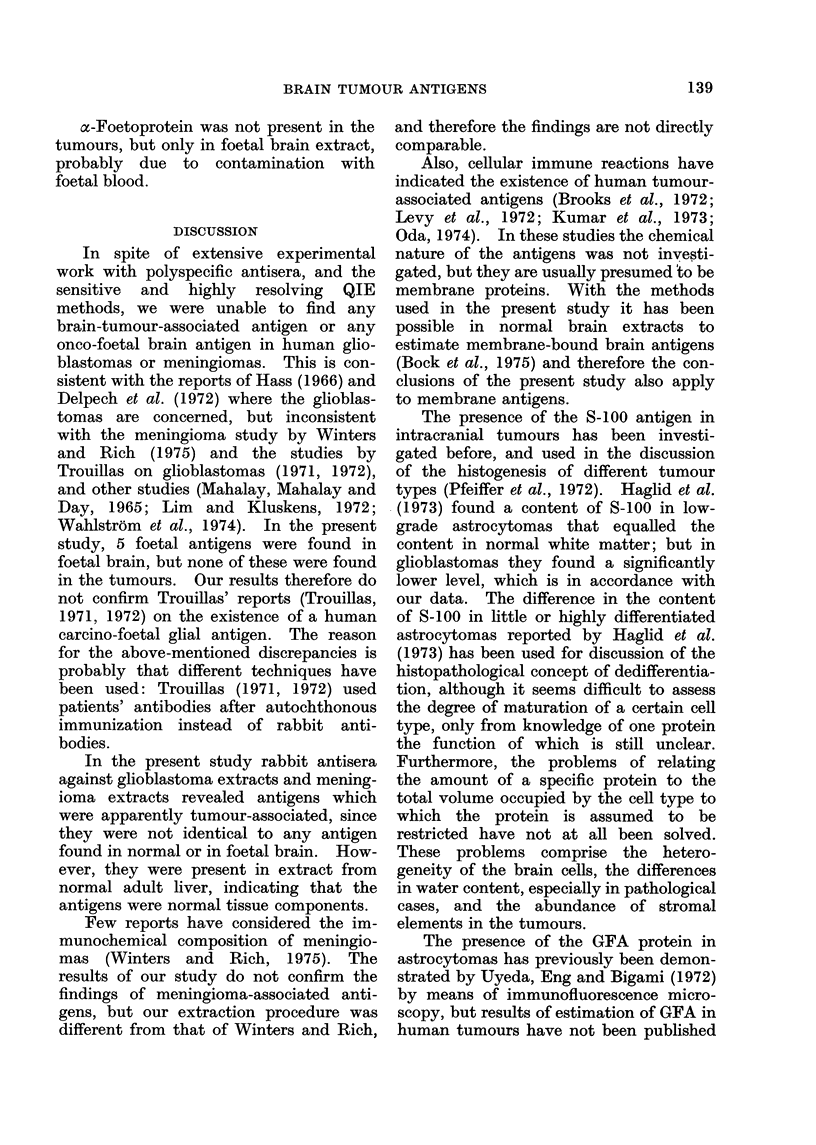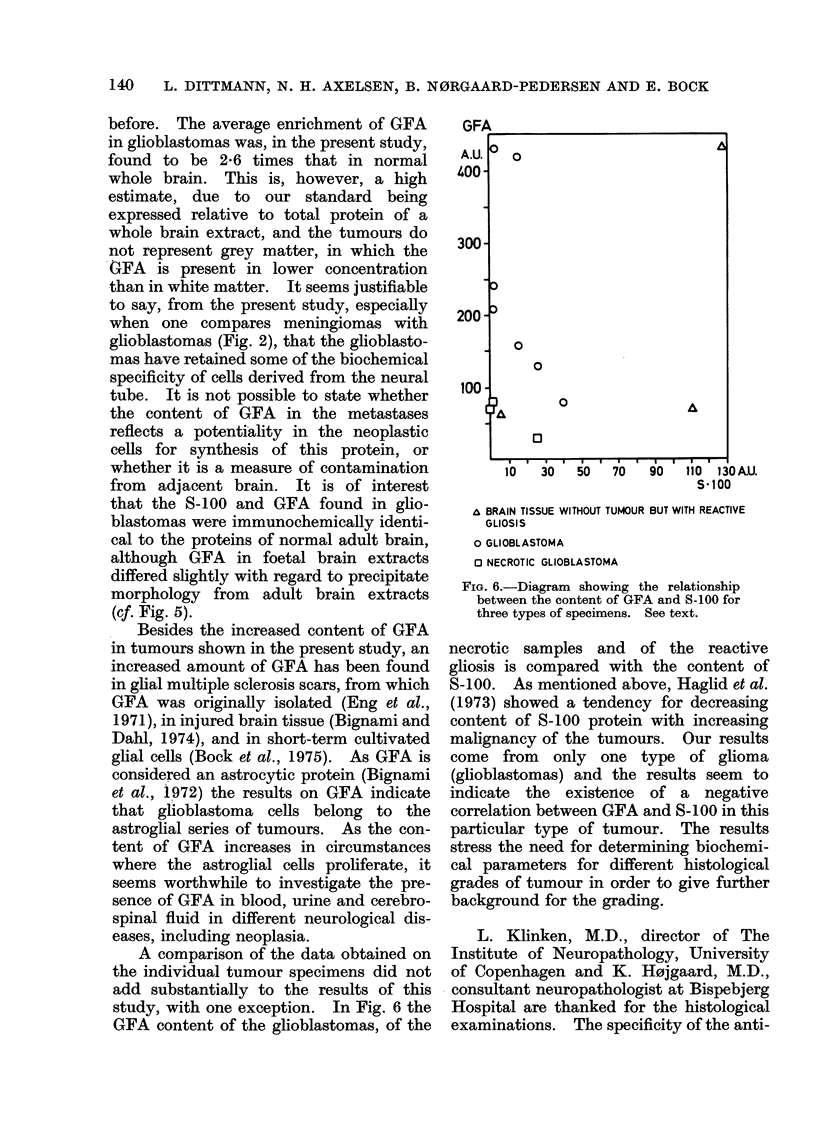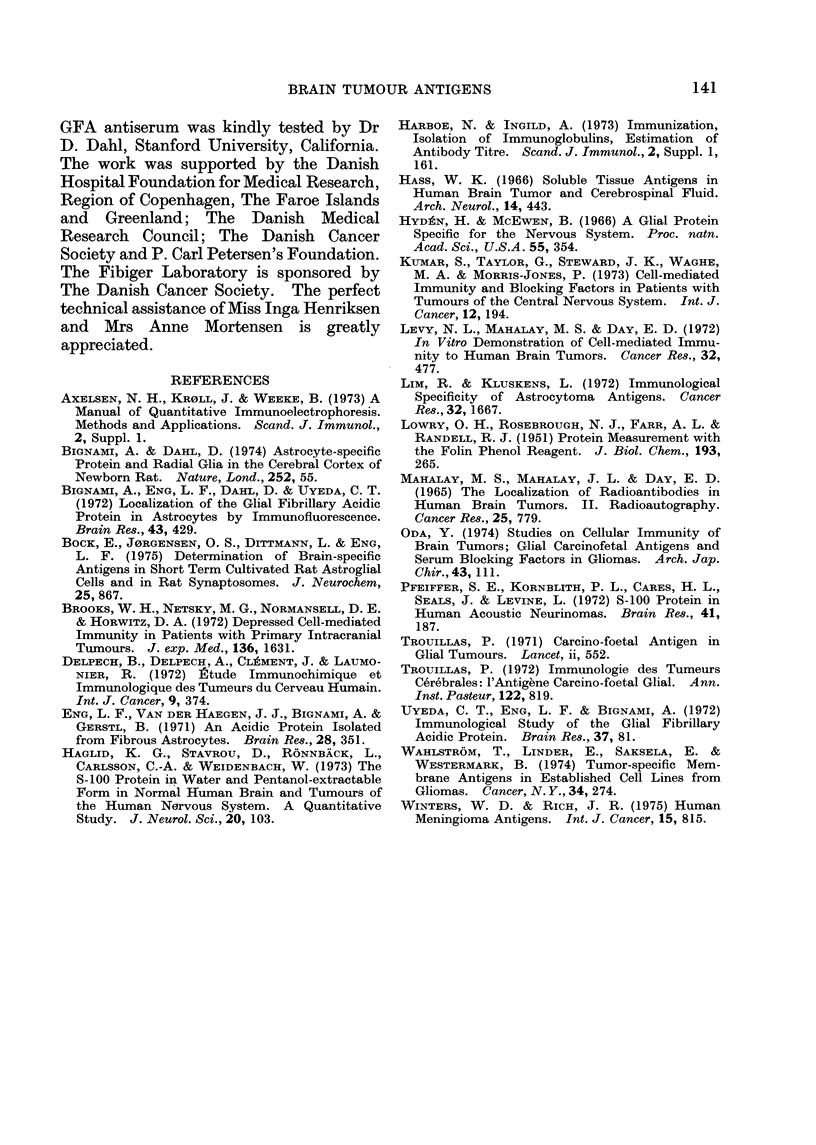# Antigens in human glioblastomas and meningiomas: Search for tumour and onco-foetal antigens. Estimation of S-100 and GFA protein.

**DOI:** 10.1038/bjc.1977.20

**Published:** 1977-02

**Authors:** L. Dittmann, N. H. Axelsen, B. Norgaard-Pedersen, E. Bock

## Abstract

**Images:**


Br. J. Cancer (1977) 35, 135

### ANTIGENS IN HUMAN GLIOBLASTOMAS AND MENINGIOMAS: SEARCH FOR TUMOUR AND ONCO-FOETAL ANTIGENS. ESTIMATION OF S-100 AND GFA PROTEIN

L. DITTMANN*, N. H. AXELSEN†, B. NØRGAARD-PEDERSEN‡ AND E. BOCK†

*From the *Bispebjerg Hospital, Dept. of Neurosurgery, DK-2400 and The Fibiger Laboratory, DK-2100 Copenhagen, Denmark, †The Protein Laboratory, University of Copenhagen, DK-2200 Copenhagen, Denmark, and ‡Dept. of Clinical Chemistry A, Rigshospitalet, DK-2100 Copenhagen, Denmark.*

Received 22 July 1976 Accepted 12 October 1976

**Summary.—Extracts of glioblastomas and meningiomas were analysed by quantitative immunoelectrophoresis for the presence of foetal brain antigens and tumour-associated antigens, and levels of 2 normal brain-specific proteins were also determined. The following antibodies were used: monospecific anti-S-100 (glia specific); monospecific anti-GFA (glial fibrillary acidic protein), (astroglia specific); polyspecific anti-foetal brain (12–16th week of gestation); a polyspecific anti-glioblastoma antiserum, absorbed with insolubilized serum, haemolysate and normal brain extract; polyspecific anti-meningioma antiserum, absorbed as for glioblastoma antiserum; monospecific anti-α-foetoprotein; and monospecific anti-ferritin. Using the antibodies raised against the tumours, several antigens not present in foetal or adult normal brain were found in the glioblastomas and the meningiomas. These antigens cross-reacted with antigens present in normal liver and were therefore not tumour-associated. S-100 was found in glioblastomas in approximately one tenth the amount in whole brain homogenate, whereas GFA was found 2–4 times enriched. The 2 proteins were absent in meningiomas. The possible use of the GFA protein as a marker for astroglial neoplasia is discussed.**

**Five foetal antigens were found in foetal brain, but none in the tumours. α-Foetoprotein could only be demonstrated in foetal tissue extracts, including foetal brain, but not in tumours. Ferritin was detected in all tumour extracts, although the amounts determined were unrelated to histological tumour type.**

In recent years, experiments have been carried out to evaluate the immune response to brain tumours in brain-tumour patients (Levy, Mahalay and Day, 1972; Oda, 1974), but few immunochemical investigations have been published on the antigenic composition of human brain tumours (Winters and Rich, 1975). The present study was undertaken to describe the antigenic composition of the two most common intracranial tumours (glioblastomas and meningiomas) by use of quantitative immunoelectrophoretic methods (Axelsen, Krøll and Weeke, 1973).

The presence or absence of neo-antigens associated with the neoplastic transformation was tested with different antisera raised in rabbits by injections of extracts of glioblastomas and meningiomas. The different tissue extracts were further analysed, using antibodies against foetal brain.

Analysis of two brain-specific antigens was performed. The glial fibrillary acidic protein (GFA) (Bignami *et al.*, 1972) is a protein located in the cytoplasm of astroglial cells. S-100 (Hydén and McEwen, 1966) is a protein predominantly located in glial cells of the brain. Finally,

Correspondence to: Nils Holger Axelsen, M.D., The Protein Laboratory, Sigurdsgade 34, DK-2200 Copenhagen, Denmark.

the amounts of ferritin were determined, and extracts were examined for the presence of $\alpha$-foetoprotein.

### MATERIALS AND METHODS

*Collection of materials and extraction of antigens.*—During operations, about 1 g of tissue was saved for immunochemical analysis. The sample was immediately transferred to dry ice and stored at $-80°C$ until analysis. Then the tumour sample was thawed and cut into pieces. One piece was sent for histological examination and the remainder was placed, together with a small metal ball, inside a nylon container fitting a Mikro-dismembrator (B. Braun, Melsungen). The nylon container was frozen in liquid $N_2$ for 4–5 min and placed in the dismembrator arm. The frozen tissue was reduced to powder by very fast movements of the metal ball inside the nylon container, which was shaken by the arm. The powder was suspended in the extraction medium (5 ml/g wet weight). The antigens were extracted with a medium consisting of 2% v/v Triton X-100, 1 mM EDTA, 15 mM $NaN_3$, 12500 kiu/l of the protease inhibitor Aprotinin (Trasylol®, Bayer) and 10 mM sodium barbital buffer adjusted to pH 8·5. The suspension was centrifuged at 180000 *g* for 45 min. This procedure was used both for the preparation of extracts for immunization of rabbits and for immunochemical analysis. Protein concentrations were determined by the method of Lowry *et al.* (1951), using bovine serum albumin as standard. To all samples, 1:10 amount of 2% w/v sodium dodecyl sulphate solution was added before total protein determination.

Normal brain was obtained from victims of traffic accidents, the autopsy being performed 8–10 h after death. Liver specimens were obtained by biopsy during gastrointestinal operations. Foetal tissues were obtained from abortions at the 12–16th week of gestation and necropsied within 1 h of abortion.

*Antibodies.*—A monospecific rabbit antiserum against human glial fibrillary acidic protein (GFA) was raised against GFA kindly supplied by Dr L. Eng, Palo Alto, California, U.S.A. A specific rabbit antiserum against bovine S-100 protein was kindly supplied by Dr K. G. Haglid, Göteborg, Sweden. Polyspecific rabbit antisera were raised against extracts of glioblastomas, meningiomas and foetal brain (12–16th week of gestation). Four rabbits were immunized with glioblastoma extracts for 1 year. Three different tumours of identical histology were used to prepare antigens and after bleeding the antisera were pooled. Three rabbits were immunized with meningioma extracts originating from 2 tumours. Immunization with foetal brain was performed on 5 rabbits for more than 1 year, using extracts of 3 foetal brains sequentially. The immunization protocol and the immunoglobulin purification method were those of Harboe and Ingild (1973).

An antiserum specific for human ferritin was a gift from Dr B. G. Johansson, Lund, Sweden.

Absorption of antisera was performed by antigens covalently linked to Sepharose using CNBr-activated Sepharose® purchased from Pharmacia, Uppsala, Sweden. One hundred and fifty milligrammes protein was coupled to 15 g Sepharose. To this, aliquots of 100 ml antiserum were absorbed, and the absorbed antibodies were eluted by 2 M guanidinium HCl, pH 3·0, before another application of antiserum. The efficiency of the absorptions was tested by crossed IE of the antigen preparations used for absorptions against the absorbed antisera.

An antiserum specific for human $\alpha$-foetoprotein was purchased from DAKO Immunoglobulins A/S, Copenhagen, Denmark.

*Quantitative immunoelectrophoresis (QIE).*—QIE (crossed IE, crossed-line IE and rocket IE) was performed as described in the manual by Axelsen, Krøll and Weeke (1973). The agarose gels contained 0·2% Triton X-100, in order to keep detergent-solubilized membrane proteins in solution. GFA, S-100 and ferritin were estimated by rocket IE, and expressed relative to total protein as per cent of the content in an extract of whole normal adult brain.

### RESULTS

Samples from 20 tumour patients and from 2 non-tumour patients were examined. A small slice of each sample was put into formalin at the time of extraction. The microscopic examination of this slice was taken as representative of the sample, and was compared with the tissue sample originally examined by the neuropathologist. Of 11 samples from operations for glioblastomas, 7 slices were well preserved, 3 showed marked necrosis, and one contained only white and grey matter with gliosis, but no tumour tissue. Two samples from non-tumour operations showing a well preserved gliosis were included in the study. Three tumours were carcinomatous metastases to the brain, all showing histological signs of contamination with adjacent brain. Six meningiomas were examined. In this way 5 categories were examined: 7 glioblastomas, 3 necrotic glioblastomas, 3 samples of gliosis, 3 metastases and 6 meningiomas. No suitable astrocytoma lower than Grade IV was found during the study. No tumour of disputable histology was included in the series.

Fig. 1 shows a crossed IE of foetal brain extract against unabsorbed polyspecific foetal brain antibodies. It was shown by crossed-line IE experiments that the foetal brain contained 5 foetal antigens (dots in Fig. 1). After sequential absorptions of the antiserum with insolubilized serum, haemolysate and adult brain, antibodies against these antigens were still present. In extracts of adult brain, adult blood, foetal liver and foetal blood, these antigens were not present. Crossed-line IE used for this purpose is able to detect foetal antigens present in tissue extracts in amounts higher than 1–5% of the amount present in the reference tissue (foetal brain). $\alpha$-Foetoprotein in Fig. 1 was identified by crossed IE with anti-$\alpha$-foetoprotein in an intermediate gel.

By crossed IE of tumour extracts or by addition of tumour extracts to foetal brain extract, it was not possible to demonstrate any of the 5 foetal antigens in any tumour extract.

Antisera against glioblastomas and against meningiomas contained more than 25 precipitating antibodies, as determined by crossed IE of tumour extracts. After absorption of the antisera with

Fig. 1.—Crossed immunoelectrophoresis (IE) of foetal brain extract (16th week of gestation) using unabsorbed anti-foetal-brain antibodies in the second dimension gel. First dimension electrophoresis at 10 V/cm for 50 min with anode at the right. Second dimension electrophoresis at 2 V/cm overnight, anode at the top. Staining: Coomassie Brilliant Blue. The dots indicate 5 foetal antigens (HF 1–HF 5). AFP, $\alpha$-foetoprotein. HSA, human serum albumin.

adult serum, haemolysate, brain and liver, all antibody activity was removed. All the individual tumour extracts were submitted to crossed IE against both the 2 absorbed tumour antisera.

The results of the estimation of GFA, S-100 and ferritin are shown in Figs. 2, 3 and 4. GFA is seen to be absent in meningiomas, close to normal in gliosis except in one case, increased 2·6 times on average in glioblastomas, but decreased in necrotic samples. S-100 was found in rather low amounts except in gliosis. Ferritin was detected in all extracts, although the amounts determined were unrelated to sample type. Analytical standard deviation of the analyses of these 3 proteins by QIE was found to be 10%.

GFA and S-100 in the glioblastomas were immunochemically identical to the normal adult GFA and S-100. GFA and S-100 were also present in foetal brain extract. Foetal S-100 was immunochemically identical to the adult protein. However, foetal GFA precipitates in a reproducibly different manner than adult GFA, indicating a physico-chemical difference (Fig. 5).

Fig. 2.—Estimation of the GFA protein. Number of samples in brackets. The content of GFA in a homogenate of whole normal adult brain is taken as 100 AU.

Fig. 3.—Estimation of the S-100 protein. Number of samples in brackets. The content of S-100 in a homogenate of whole normal adult brain is taken as 100 AU.

Fig. 4.—Estimation of ferritin. Number of samples in brackets. The content of ferritin in a homogenate of whole normal adult brain is taken as 100 AU.

Fig. 5.—Fused rocket immunoelectrophoresis of extracts of adult brain (a, 4 μl containing 9·2 μg protein) and foetal brain (b, 20 μl containing 348 μg protein). Antiserum is anti-GFA antiserum, 2·5 μl/cm². GFA in both extracts shows reaction of immunochemical identity; however the foetal GFA precipitate is more blurred than the adult GFA precipitate, indicating physico-chemical differences.

α-Foetoprotein was not present in the tumours, but only in foetal brain extract, probably due to contamination with foetal blood.

### DISCUSSION

In spite of extensive experimental work with polyspecific antisera, and the sensitive and highly resolving QIE methods, we were unable to find any brain-tumour-associated antigen or any onco-foetal brain antigen in human glioblastomas or meningiomas. This is consistent with the reports of Hass (1966) and Delpech *et al.* (1972) where the glioblastomas are concerned, but inconsistent with the meningioma study by Winters and Rich (1975) and the studies by Trouillas on glioblastomas (1971, 1972), and other studies (Mahalay, Mahalay and Day, 1965; Lim and Kluskens, 1972; Wahlström *et al.*, 1974). In the present study, 5 foetal antigens were found in foetal brain, but none of these were found in the tumours. Our results therefore do not confirm Trouillas' reports (Trouillas, 1971, 1972) on the existence of a human carcino-foetal glial antigen. The reason for the above-mentioned discrepancies is probably that different techniques have been used: Trouillas (1971, 1972) used patients' antibodies after autochthonous immunization instead of rabbit antibodies.

In the present study rabbit antisera against glioblastoma extracts and meningioma extracts revealed antigens which were apparently tumour-associated, since they were not identical to any antigen found in normal or in foetal brain. However, they were present in extract from normal adult liver, indicating that the antigens were normal tissue components.

Few reports have considered the immunochemical composition of meningiomas (Winters and Rich, 1975). The results of our study do not confirm the findings of meningioma-associated antigens, but our extraction procedure was different from that of Winters and Rich, and therefore the findings are not directly comparable.

Also, cellular immune reactions have indicated the existence of human tumour-associated antigens (Brooks *et al.*, 1972; Levy *et al.*, 1972; Kumar *et al.*, 1973; Oda, 1974). In these studies the chemical nature of the antigens was not investigated, but they are usually presumed to be membrane proteins. With the methods used in the present study it has been possible in normal brain extracts to estimate membrane-bound brain antigens (Bock *et al.*, 1975) and therefore the conclusions of the present study also apply to membrane antigens.

The presence of the S-100 antigen in intracranial tumours has been investigated before, and used in the discussion of the histogenesis of different tumour types (Pfeiffer *et al.*, 1972). Haglid *et al.* (1973) found a content of S-100 in low-grade astrocytomas that equalled the content in normal white matter; but in glioblastomas they found a significantly lower level, which is in accordance with our data. The difference in the content of S-100 in little or highly differentiated astrocytomas reported by Haglid *et al.* (1973) has been used for discussion of the histopathological concept of dedifferentiation, although it seems difficult to assess the degree of maturation of a certain cell type, only from knowledge of one protein the function of which is still unclear. Furthermore, the problems of relating the amount of a specific protein to the total volume occupied by the cell type to which the protein is assumed to be restricted have not at all been solved. These problems comprise the heterogeneity of the brain cells, the differences in water content, especially in pathological cases, and the abundance of stromal elements in the tumours.

The presence of the GFA protein in astrocytomas has previously been demonstrated by Uyeda, Eng and Bigami (1972) by means of immunofluorescence microscopy, but results of estimation of GFA in human tumours have not been published before. The average enrichment of GFA in glioblastomas was, in the present study, found to be 2·6 times that in normal whole brain. This is, however, a high estimate, due to our standard being expressed relative to total protein of a whole brain extract, and the tumours do not represent grey matter, in which the GFA is present in lower concentration than in white matter. It seems justifiable to say, from the present study, especially when one compares meningiomas with glioblastomas (Fig. 2), that the glioblastomas have retained some of the biochemical specificity of cells derived from the neural tube. It is not possible to state whether the content of GFA in the metastases reflects a potentiality in the neoplastic cells for synthesis of this protein, or whether it is a measure of contamination from adjacent brain. It is of interest that the S-100 and GFA found in glioblastomas were immunochemically identical to the proteins of normal adult brain, although GFA in foetal brain extracts differed slightly with regard to precipitate morphology from adult brain extracts (*cf.* Fig. 5).

Besides the increased content of GFA in tumours shown in the present study, an increased amount of GFA has been found in glial multiple sclerosis scars, from which GFA was originally isolated (Eng *et al.*, 1971), in injured brain tissue (Bignami and Dahl, 1974), and in short-term cultivated glial cells (Bock *et al.*, 1975). As GFA is considered an astrocytic protein (Bignami *et al.*, 1972) the results on GFA indicate that glioblastoma cells belong to the astroglial series of tumours. As the content of GFA increases in circumstances where the astroglial cells proliferate, it seems worthwhile to investigate the presence of GFA in blood, urine and cerebrospinal fluid in different neurological diseases, including neoplasia.

A comparison of the data obtained on the individual tumour specimens did not add substantially to the results of this study, with one exception. In Fig. 6 the GFA content of the glioblastomas, of the necrotic samples and of the reactive gliosis is compared with the content of S-100. As mentioned above, Haglid *et al.* (1973) showed a tendency for decreasing content of S-100 protein with increasing malignancy of the tumours. Our results come from only one type of glioma (glioblastomas) and the results seem to indicate the existence of a negative correlation between GFA and S-100 in this particular type of tumour. The results stress the need for determining biochemical parameters for different histological grades of tumour in order to give further background for the grading.

FIG. 6.—Diagram showing the relationship between the content of GFA and S-100 for three types of specimens. See text.

L. Klinken, M.D., director of The Institute of Neuropathology, University of Copenhagen and K. Højgaard, M.D., consultant neuropathologist at Bispebjerg Hospital are thanked for the histological examinations. The specificity of the anti-

GFA antiserum was kindly tested by Dr D. Dahl, Stanford University, California. The work was supported by the Danish Hospital Foundation for Medical Research, Region of Copenhagen, The Faroe Islands and Greenland; The Danish Medical Research Council; The Danish Cancer Society and P. Carl Petersen's Foundation. The Fibiger Laboratory is sponsored by The Danish Cancer Society. The perfect technical assistance of Miss Inga Henriksen and Mrs Anne Mortensen is greatly appreciated.

### REFERENCES

AXELSEN, N. H., KRØLL, J. & WEEKE, B. (1973) A Manual of Quantitative Immunoelectrophoresis. Methods and Applications. *Scand. J. Immunol.*, **2**, Suppl. 1.

BIGNAMI, A. & DAHL, D. (1974) Astrocyte-specific Protein and Radial Glia in the Cerebral Cortex of Newborn Rat. *Nature, Lond.*, **252**, 55.

BIGNAMI, A., ENG, L. F., DAHL, D. & UYEDA, C. T. (1972) Localization of the Glial Fibrillary Acidic Protein in Astrocytes by Immunofluorescence. *Brain Res.*, **43**, 429.

BOCK, E., JØRGENSEN, O. S., DITTMANN, L. & ENG, L. F. (1975) Determination of Brain-specific Antigens in Short Term Cultivated Rat Astroglial Cells and in Rat Synaptosomes. *J. Neurochem*, **25**, 867.

BROOKS, W. H., NETSKY, M. G., NORMANSELL, D. E. & HORWITZ, D. A. (1972) Depressed Cell-mediated Immunity in Patients with Primary Intracranial Tumours. *J. exp. Med.*, **136**, 1631.

DELPECH, B., DELPECH, A., CLÉMENT, J. & LAUMONIER, R. (1972) Étude Immunochimique et Immunologique des Tumeurs du Cerveau Humain. *Int. J. Cancer*, **9**, 374.

ENG, L. F., VAN DER HAEGEN, J. J., BIGNAMI, A. & GERSTL, B. (1971) An Acidic Protein Isolated from Fibrous Astrocytes. *Brain Res.*, **28**, 351.

HAGLID, K. G., STAVROU, D., RÖNNBÄCK, L., CARLSSON, C.-A. & WEIDENBACH, W. (1973) The S-100 Protein in Water and Pentanol-extractable Form in Normal Human Brain and Tumours of the Human Nervous System. A Quantitative Study. *J. Neurol. Sci.*, **20**, 103.

HARBOE, N. & INGILD, A. (1973) Immunization, Isolation of Immunoglobulins, Estimation of Antibody Titre. *Scand. J. Immunol.*, **2**, Suppl. 1, 161.

HASS, W. K. (1966) Soluble Tissue Antigens in Human Brain Tumor and Cerebrospinal Fluid. *Arch. Neurol.*, **14**, 443.

HYDÉN, H. & MCEWEN, B. (1966) A Glial Protein Specific for the Nervous System. *Proc. natn. Acad. Sci., U.S.A.* **55**, 354.

KUMAR, S., TAYLOR, G., STEWARD, J. K., WAGHE, M. A. & MORRIS-JONES, P. (1973) Cell-mediated Immunity and Blocking Factors in Patients with Tumours of the Central Nervous System. *Int. J. Cancer*, **12**, 194.

LEVY, N. L., MAHALAY, M. S. & DAY, E. D. (1972) *In Vitro* Demonstration of Cell-mediated Immunity to Human Brain Tumors. *Cancer Res.*, **32**, 477.

LIM, R. & KLUSKENS, L. (1972) Immunological Specificity of Astrocytoma Antigens. *Cancer Res.*, **32**, 1667.

LOWRY, O. H., ROSEBROUGH, N. J., FARR, A. L. & RANDELL, R. J. (1951) Protein Measurement with the Folin Phenol Reagent. *J. Biol. Chem.*, **193**, 265.

MAHALAY, M. S., MAHALAY, J. L. & DAY, E. D. (1965) The Localization of Radioantibodies in Human Brain Tumors. II. Radioautography. *Cancer Res.*, **25**, 779.

ODA, Y. (1974) Studies on Cellular Immunity of Brain Tumors; Glial Carcinofetal Antigens and Serum Blocking Factors in Gliomas. *Arch. Jap. Chir.*, **43**, 111.

PFEIFFER, S. E., KORNBLITH, P. L., CARES, H. L., SEALS, J. & LEVINE, L. (1972) S-100 Protein in Human Acoustic Neurinomas. *Brain Res.*, **41**, 187.

TROUILLAS, P. (1971) Carcino-foetal Antigen in Glial Tumours. *Lancet*, ii, 552.

TROUILLAS, P. (1972) Immunologie des Tumeurs Cérébrales: l'Antigène Carcino-foetal Glial. *Ann. Inst. Pasteur*, **122**, 819.

UYEDA, C. T., ENG, L. F. & BIGNAMI, A. (1972) Immunological Study of the Glial Fibrillary Acidic Protein. *Brain Res.*, **37**, 81.

WAHLSTRÖM, T., LINDER, E., SAKSELA, E. & WESTERMARK, B. (1974) Tumor-specific Membrane Antigens in Established Cell Lines from Gliomas. *Cancer, N.Y.*, **34**, 274.

WINTERS, W. D. & RICH, J. R. (1975) Human Meningioma Antigens. *Int. J. Cancer*, **15**, 815.